# Therapeutic Alliance in Cognitive Behavioural Therapy in Child and Adolescent Mental Health-Current Trends and Future Challenges

**DOI:** 10.3389/fpsyg.2021.610874

**Published:** 2022-01-03

**Authors:** Hazel Fernandes

**Affiliations:** Child and Adolescent Psychiatry, Health Service Executive, Dublin, Ireland

**Keywords:** child and adolescent mental health, therapeutic alliance, cognitive behavioural therapy, literature review, current trends

## Abstract

This extended literature review proposes to present the trends in the therapeutic alliance, outcomes, and measures in the last decade within the premises of individual cognitive behaviour therapy (CBT) and its innovations, used as an interventional measure in the context of child and adolescent mental health setting. A brief background of the rationale for conducting this literature search is presented at the start. This is followed by the methodology and design which incorporates the inclusion and exclusion criteria and the basis for the same. The critical appraisal of the primary studies is presented in the literature review section with a brief description of the summary features of the studies in the study tables followed by the results and discussion of the study findings. To summarise, the literature review of primary studies conducted in the last decade demonstrates the need for further research to be conducted both in the field of CBT in children and therapeutic alliance, competence, and therapy outcomes, integrating perspectives in child development, carer alliance, and the social construct theory in children, to allow for further innovations in CBT in the context of increasing challenges in the current times of exponentially developing technology and its utility without compromising the quality of therapy. In conclusion, recommendations are made as a guideline for future studies and research in this field.

## Introduction

### Background and Rationale

In Psychotherapeutic settings, the relationship between the clinician and the client is essential and although complex, it is an important factor for effective practice. Outcomes in therapy have been consistently related to the therapeutic alliance (Arnow and Steidtmann, 2014). Bachelor (2013) concluded that the view of clients towards the therapeutic process significantly influenced the treatment outcome and therefore, recommended seeking client feedback to achieve superior treatment outcomes. The conditions need to be optimised and, in therapy, both the therapist and client need to collaborate, innovate, and re-evaluate goals and find common grounds to allow for the achievement of goals agreed at the beginning of therapy.

Cognitive behavioural therapy (CBT), traditionally seen as manual-based therapy, allows for the little opportunity for the development of a therapeutic relationship. In their book, Sanders and Wills (1999) expressed the view that therapists drawn to CBT tended to lose interest in it due to this lack of emphasis on the therapeutic relationship. On the other hand, others reported that too much emphasis was placed on “the technical aspects of therapy,” rather than focusing on the perception of the client on the “relationship with the therapist” (Duncan et al., 2010). Wolfe and Goldfried (1988) called a therapeutic alliance, “the quintessential integrative variable” of therapy. Norcross (2010) defined the therapeutic alliance as referring to “the quality and strength of the collaborative relationship between client and therapist, typically measured as agreement on the therapeutic goals, consensus on treatment tasks, and a relationship bond.”

The changes in the “variations” during the different phases of therapy were attributed to the changes in “alliance,” while proposing a theoretical concept of alliance (Luborsky,1976). However, Bowlby (1988) theorised that how the patient behaved in his attachment towards the therapist was qualitatively based on the childhood experiences of the patient (Bowlby, 1988). A distinction was made between transference and alliance and propagated beyond the psychoanalytic framework (Bowlby, 1988; Horvath and Luborsky, 1993).

Bordin coined the term, “working alliance,” to define this relationship between client and therapist which he postulated and negotiated. Although he did not emphasise client contribution and preferred to place the onus on the therapist to perform, he felt that it was not a positive alliance, but a working alliance, that was key to the therapy being successful (Bordin, 1976, 1979, 1980, 1994). This contrasted with the opinion of Rogners that clients automatically respond to the positive attitude of the therapist (Rogers, 1951). Carl Rogers stated that there are “three vital components” that constitute therapeutic alliance: empathy, congruence, and unconditional positive regard (Rogers, 1951). The therapeutic alliance is based on the foundation of collaboration, bond formation, and common goals (Ardito and Rabellino, 2011). A meta-analytic study in adults indicated that therapeutic alliance was the strongest predictor of psychotherapy drop-out (Sharf et al., 2010).

### Objectives of the Literature Review

To determine the role of the developmental age of the child and social constructs of the child’s relationship with therapeutic alliance.

To determine if the alliance with carers is a significant predictor of the therapeutic alliance with a child and if this predicts treatment outcome.

To determine the validity of using the Psychometric rating scales to assess therapeutic alliance in children.

To evaluate the effect of therapist factors with respect to training and supervision on alliance and treatment outcome is another area of interest of this review.

To determine the effectiveness of Telemental health interventions with respect to CBT and the effectiveness of these methods to establish an alliance in children.

To evaluate the effect of medication on therapeutic alliance.

The hypothesis is that developmental age and social constructs of the relationship patterns of a child will play a significant role in establishing therapeutic alliance and will demonstrate that establishing an alliance with carers is a very significant part of the therapeutic alliance with a child, significantly affecting therapeutic alliance. The regular supervision of a therapist improves therapeutic alliance, in turn, influencing treatment outcomes. It is possible to establish effective therapeutic alliances through online CBT use.

## Materials and Methods

### The Integrative Review

The integrative method allows for an overview of therapeutic alliance studies that are more diverse, both experimental and non-experimental. It includes systematic categorisation and thematic analysis of selected research studies, both qualitative and quantitative studies, randomised controlled trials (RCTs), observational studies, or any other relevant evidence to draw inference about a topic (Whittemore and Knafl, 2005). This will allow for a more comprehensive understanding to provide the answers we hope to find and help to synthesise the knowledge gained to perhaps amalgamate this into new recommendations and proposals. Objectively critiquing, summarising, and drawing inferences will help the thematic analysis of the literature being studied (Silveira, 2005). This includes systematic categorisation and thematic analysis of selected qualitative and quantitative research studies, to generate new interpretations from the analysis of the studies (Dixon-Woods et al., 2005).

The integrative review will review the current outcomes from research and this, in turn, will help in understanding the current trends in Therapeutic Alliance in Child and Adolescent Mental Health (Cooper, 1998).

### Limitations of the Integrative Review

The five-stage integrative review process includes (1) problem formulation, (2) data collection or literature search, (3) evaluation of data, (4) data analysis, and (5) interpretation and presentation of results (Whittemore and Knafl, 2005). One of the difficulties is mastering the sophistication and adherence to detail that is essential to the integrative review methodology (Torraco, 2005). Although the integrative review is limited to published literature, it facilitates the conceptualisation of variables being studied. In addition, until recently, statistical procedures were not applied to the studies reviewed, and several strategies ranging from the robust application of inclusion and exclusion criteria to commenting on demographics were suggested as measures to increase the validity of these reviews (Cooper, 1998; Russell, 2005).

### Information Sources

The EBSCOhost Research Database was used to search in MEDLINE, CINAHL Plus with Full Text, Health Business Elite, Library, Information Science and Technology Abstracts, and APA PsycInfo. Google Scholar was also used. The snowball method (Lecy and Beatty, 2012) was also utilised. Information was obtained through online electronic searches from peer-reviewed journals, refereed articles, dissertations, case reports, and opinion pieces.

### Study Selection Criteria

Quality Assessment of the studies included was done by using The Critical Appraisal Skills Programme (2018) checklist for assessing the studies included in the literature review (Singh, 2013).

### Google Scholar Search

Google Scholar search was conducted using the same terms as in the EBSCOHost Research Database search and the first 50 results displayed by relevance were checked. Only two search results had not been figured in the EBSCOHost search, and these were added to the 432 results obtained from the EBSCOHost search for further review to check if they met the inclusion criteria.


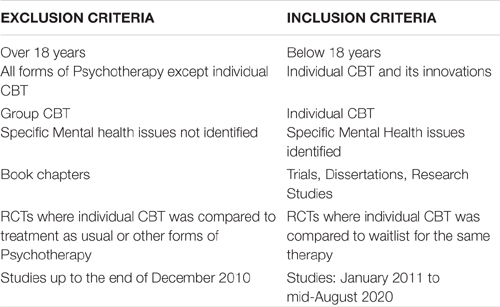


### Search Terms

#### Keywords

Therapeutic Alliance, CBT, child, adolescent.

**FIGURE 1:**
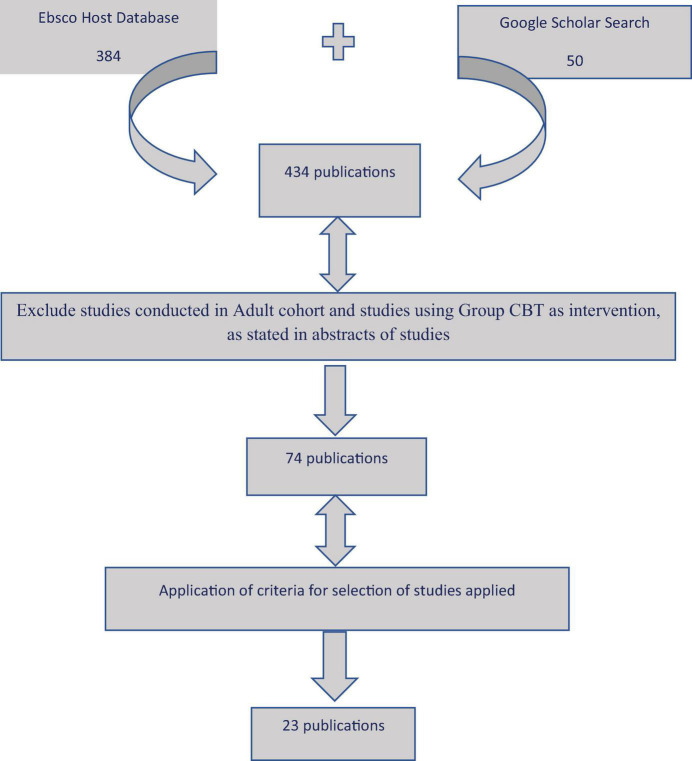
Flow diagram of search of databases.

George Boole, a 19th-century English mathematician, developed a method of symbolic logic. Boolean searches on the database allowed the combination of words to allow the search to be limited to those studies that had either therapeutic alliance and cognitive behavioural therapy and child. It helped to broaden the search by adding the words Therapy* alliance, child* and cognitive behavioural* therapy (“Behaviour” and “behavior” as it is spelt differently across the world by a different researcher). I did not include the word “not,” to restrict the search, as I hoped to read through search results to determine if any of the studies progressed from adolescence to adulthood, with respect to studying therapeutic alliance. The initial search carried out on the 12 April 2020 yielded 381 results and, after removing duplicates, 250 were left. Another search that was carried out in June yielded the same results. The final search was carried out on the 12 August About 74 results were selected for further review for an in-depth screening of the full articles to make sure they met all the inclusion criteria, and 23 studies met all the inclusion criteria (Figure 1 and Supplementary Table 1).

## Results

### A-Study Features

#### Lead Authors of Studies

All the lead authors of the studies were Psychologists by orientation.

#### Countries

The studies were conducted in the United States of America (13), Canada (3), Australia (1) Germany (3), Denmark (1), Netherlands (1), and Norway (1).

#### Language

All but one of the publications were in English. The foreign language research was also translated to Spanish and Chinese within the same study.

#### Publications

The impact factor ranged from the lowest at 1.622 (Journal: Psychotherapy) to the highest at 5.879 (Autism: The International Journal of Research and Practice). The average impact factor of the publications was 3.255.

#### Database

APA PsycInfo:17, MEDLINE:5, Open Access: 1.

#### Study Type

Cohort study: 11 (Including Study 2 from Anderson et al., 2012).

RCT:13- Including Study 1 from Anderson et al. (2012).

Randomisation: The participants in the RCT studies were randomised using appropriate and statistically sound randomisation methods. One study stated that the researchers conducting the randomisation were aware of the treatment assignment (Klebanoff et al., 2019).

The observers and coders who rated either video or audiotaped sessions were blinded to the study hypotheses (Hudson et al., 2014; Boyer et al., 2018; Kirsch et al., 2018; Puls et al., 2019).

#### Ethics and Confidentiality

All studies obtained relevant ethics approvals from their institution ethics board or research ethics boards.

Confidentiality of patient or parent ratings maintained during the study were specifically mentioned in three studies (Keeley et al., 2011; Marker et al., 2013; McLeod et al., 2017).

#### Selection of Study Participants

The recruitment methods for the studies differed. The majority of the studies recruited participants and families from specialty clinics that patients attended for specific disorders. Anderson et al. (2012) recruited through public media campaigns and requests to health professionals. Albaum et al. (2020) did not explicitly state the site of recruitment but stated that the study subjects were part of a larger RCT. Other studies recruited subjects that were referred by school personnel through the invitation to schools and the public (Whitehead et al., 2019) or by referrals from schools (Labouliere et al., 2017). In one study, families were provided with a $15 moratorium to participate in the pre- and post-treatment assessments (Klebanoff et al., 2019). Labouliere et al. (2017) used a more real-life clinical setting for their study.

#### Sample Size

Sample size ranged in number from the lowest, specifically, 4 and 13 participants (Valadez-Sanchez, 2017; Carpenter et al., 2018) to 151 participants (Hudson et al., 2014). The majority of the studies had between 50 and 100 participants.

#### Demographics

##### Age

The age range of participants was between 5 and 18 years. One study had 7 participants who were 20 years old. In this study, the participants who were 18-years-old at the commencement of intervention after being recruited were included in the study as their living conditions were comparable to those at 18 years of age (Puls et al., 2019). The average age for the studies included was 12 years.

##### Sex

Both males and females were included in the studies. Labouliere et al. (2017) had nearly twice the number of females compared to males. Females outnumbered males in the Trauma studies by a nearly 5:1 ratio (Zorzella et al., 2015, 2017). The Valadez-Sanchez study had four female participants (Valadez-Sanchez, 2017). Male participants were 91.7% of the study participants in the study on ADHD (Albaum et al., 2020).

##### Ethnicity

The majority of the study subjects were Caucasian but the study sample of Labouliere et al. (2017) was relatively diverse in terms of race and ethnicity. All four participants in the Valadez-Sanchez study were Latina females who fulfilled the study criteria (Valadez-Sanchez, 2017).

##### Socioeconomic status

The majority of the study participants in the anxiety related studies were of higher socioeconomic status and moderately higher educational status. The socioeconomic status of the study population was at the lower end of the scale in those that involved trauma and related disorders (Zorzella et al., 2015, 2017). The other study involving PTSD did not elaborate on socioeconomic status.

#### Measures of Therapeutic Alliance

TABBS Therapist Alliance-Building Behaviour Scale.

TASC/TASC-r (for children and caregivers).

TPOCS-A Therapy Process Observational Coding System-Alliance Scale.

VTAS Vanderbilt Therapeutic Alliance Scale.

WAI-Working Alliance Inventory.

#### Mental Health Issues

Studies were conducted in the context of Attention Deficit Hyperactivity Disorder (ADHD), Autism, binge eating disorder (BED), Depressive disorder, Obsessive Compulsive Disorder (OCD), Trauma, and Post-traumatic Stress Disorder (PTSD).

The occurrence of comorbid mental health issues ranged from about 60% (Marker et al., 2013), 90% (Zandberg et al., 2015), and 92% (Whitehead et al., 2019) of the participants.

#### Medication

Medication was continued throughout the study, although it required the patients to be on a stable dose for an average of about 4 weeks. In one study on children with anxiety disorder, 42% were on a selective serotonin uptake inhibitor (SSRI) (Chu et al., 2014). In the only study on children with ADHD, 83% were on medication (Boyer et al., 2018).

#### Type of Cognitive Behaviour Therapy Interventions

Individual CBT was the predominant intervention used.

Family based CBT was used in three studies, with two studies that used clinic based (Keeley et al., 2011; Marker et al., 2013), and one that used TeleMental Health Family based CBT (Carpenter et al., 2018). Anderson et al. (2012) randomised participants to individual CBT and internet-based CBT. Internet-based CBT (Stjerneklar et al., 2019), Multimodal CBT-(Klebanoff et al., 2019), Trauma focused CBT- (Zorzella et al., 2015, 2017; Valadez-Sanchez, 2017; Kirsch et al., 2018; Loos et al., 2020), and Emotion regulation focused CBT was used by Albaum et al. (2020).

### B-Study Aims

#### Obsessive Compulsive Disorder

The aim was to study the influence of therapeutic alliance ratings of the therapist, client, and parent on the later reduction in OCD symptoms and whether the strength of the alliance itself was predictive of treatment outcome as indicated by a reduction in OCD symptoms (Keeley et al., 2011).

#### Anxiety Disorder

Internet-based family based CBT was studied with respect to variables like Therapeutic Alliance (Carpenter et al., 2018).

Anderson et al. (2012) attempted to determine if internet-based CBT affected the quality of working alliance between Clinic based and internet-based CBT intervention. In addition, the study aims in one study were to determine the effect of alliance and compliance on treatment outcome and furthermore, if this compliance affects the relationship between working alliance and treatment outcome in the context of online CBT (Anderson et al., 2012).

Patient demographics and clinical and therapy related predictors (including Therapeutic Alliance) were studied with respect to treatment outcome using Internet-based CBT in another study (Stjerneklar et al., 2019).

Levin et al. (2012), aimed to identify factors predicting early alliance in adolescents with anxiety and/or depressive disorders.

Chu et al. (2014) focused on the changes in the direction of therapeutic alliance throughout therapy and the treatment factors that influence the trajectories with the treatment factors being: the effect of age and sex, symptom severity before the intervention, comorbidity, adjunctive medication, pre-treatment coping styles were also studied.

Zandberg et al. (2015) aimed to study the relationship between the youth alliance ratings and the therapists rating of therapeutic alliance at different points during therapy and if the changes predicted treatment outcome.

The quality of child-therapist alliance predicting the extent of in-session involvement and vice versa and the relationship of the two variables was evaluated by McLeod et al. (2014). McLeod et al., also evaluated the score and observer validity of the various alliance scales used to measure Therapeutic Alliance (McLeod et al., 2017).

Whitehead et al. (2019), sought to evaluate if Therapeutic Alliance was predicted by the pre-treatment social and emotional behaviours of a child.

##### Treatment outcome studies

Studies set out to determine if one variable, i.e., compliance with collaborated tasks predicted treatment outcome (reduction of symptoms) and if there was improved therapeutic alliance due to reduction of anxiety symptoms (Marker et al., 2013). Particularly, if the relationship of any variables, i.e., child and therapist alliance, functionality, and flexibility of the child and therapist relationship of the treatment processes related to the treatment outcome (Hudson et al., 2014). Finally, if the quality of the child-therapist alliance predicted the treatment outcome (Fjermestad et al., 2016).

#### Depressive Disorder

The evaluation of the trajectory of the therapeutic alliance with respect to depressive symptomatology and the influence of these symptoms on the therapeutic alliance was the aim of one study (Labouliere et al., 2017).

#### Trauma and Post Traumatic Stress Disorder

Trauma studies aimed to study:

The effect of pre-treatment symptomatology on early Therapeutic alliance and if treatment outcome is predicted by this alliance (Zorzella et al., 2015).

Alliance changes through multiple ratings of different patients when the trauma narrative is introduced in Trauma-focused CBT (TF-CBT) (Zorzella et al., 2017).

Valadez-Sanchez (2017), sought to understand how Culturally Modified Trauma-Focused Treatment (CM-TFT) modified alliance, engagement with intervention, and post-treatment symptomatology.

Treatment expectation effects and the effects of child-therapist collaboration and parent working alliance affected treatment outcome (Kirsch et al., 2018). Post-traumatic stress symptoms reduction was associated with Therapeutic alliance (Loos et al., 2020).

#### Attention Deficit Hyperactivity Disorder

The ADHD study sought to compare the quality of alliance between variations of CBT, a structured planning implementation module (PML), and a less structured solution-focused treatment (SFT). It also aimed to determine if treatment outcome was determined by the difference in the alliance between the two treatments and further if alliance predicted the relationship between therapist competence and treatment outcome (Boyer et al., 2018).

#### Eating Disorder

The utility of the therapist adherence form (ACF) used in adults to measure adherence and alliance in children study patient and therapist factors influencing adherence, the variability of the alliance, and the association between adherence and alliance at different points during treatment and 6 and 12 months post-treatment. All of which were the study aims (Puls et al., 2019).

#### Autism

Two studies sought to compare two cohorts. One was with autism spectrum disorder (ASD) and anxiety and the other cohort with anxiety only. The study sought to compare the strength of therapeutic alliance in each, utilising the intervention of Multimodal Cognitive Behavioural Therapy (MCBT; Klebanoff et al., 2019) while (Albaum et al., 2020) aimed to study if the therapeutic alliance was significantly predicted by pre-treatment symptomatology and whether early or late therapeutic alliance would implement a change in the emotional regulation using emotion regulation focussed CBT.

### C-Study Conclusion

#### Obsessive Compulsive Disorder

There was a significant relationship between therapist-rated alliance and symptom reduction in that the strength of the ratings of all three raters (child, parent, and therapist) directly related to the decrease in symptomatology. However, there was a significant decrease observed in parent rating of the therapeutic alliance towards the latter part of the intervention. Hence, the exposure work did not impact the ability of the therapeutic alliance measure to predict treatment outcomes (Keeley et al., 2011).

#### Anxiety Disorder

High alliance rates and treatment adherence was found with completion rates of more than 80% in the online CBT intervention (Carpenter et al., 2018). There was no difference in online CBT alliance ratings and face-to-face CBT alliance ratings of both parent and child in the study by Anderson et al. (2012), but the parent-rated alliance was significantly higher than their counterparts in the face-to-face CBT. Furthermore, although the anxiety symptoms improved significantly, these changes were not predicted by the working alliance even though the working alliance predicted youth alliance significantly. Compliance in the treatment did not significantly predict the outcome, but parents and youth alliance were significantly related (Anderson et al., 2012).

There was a significant association between parent, child, and therapist ratings of alliance and youth reporting higher rates of supportive relationships and established stronger alliances (Levin et al., 2012).

Although not statistically significant, the findings from another study demonstrated that social functioning and emotional regulation ability were related to the therapeutic alliance as rated in children and teacher report forms (Whitehead et al., 2019).

##### Anxiety treatment outcome and alliance

The alliance ratings of therapists and mothers had a predictive effect on improvement in anxiety symptoms. In contrast, ratings of the alliance by fathers and children did not predict improvement in anxiety symptoms. In view of this finding, it was surprising to find that, with respect to reverse causality, it was the alliance ratings of the fathers and therapists that were predicted by the reduction in anxiety symptoms and not the children and mothers (Marker et al., 2013).

Hudson et al. (2014), found that therapist involvement directly influences child alliance and involvement and teachers observed a decrease in symptoms when therapeutic alliance improved.

Despite this, no significant differences were found between therapist and child-rated alliance scores and this did not significantly predict treatment outcome (Zandberg et al., 2015).

Concurrence of therapist and patient agreement about alliance ratings positively predicted treatment outcome. The higher early alliance score led to higher patient satisfaction as measured post-treatment (Fjermestad et al., 2016). Therapeutic alliance did not predict treatment response in the study using online CBT (Stjerneklar et al., 2019).

Baseline severity of symptoms, as rated by both patients and therapists, predicted treatment response (Levin et al., 2012; Stjerneklar et al., 2019).

When high and low alliance were plotted as a function of each of the variables of “Pre-treatment depression and anxiety severity” and “engagement coping,” the “Dual slope” model was more efficient in demonstrating the alliance trajectory (Chu et al., 2014). The study also found that externalising problems did not have a significant effect on alliance (Chu et al., 2014).

One study suggested that from their findings, the participation of the clients in therapy was strongly related to the collaboration with the therapist and the affective quality of that relationship both in early and late in therapy (McLeod et al., 2014). This further suggests that the involvement was significantly directly proportional to age. That is, the older the child, the stronger was the involvement and the level of involvement was influenced by an increase in the alliance but not vice-versa (McLeod et al., 2014).

Another study also found no evidence of exposure sessions risking client alliance and engagement in anxiety disorder and instead observed that alliance rating improvements continued throughout treatment (Chu et al., 2014).

#### Depressive Disorder

Early therapeutic alliance significantly influenced the latter severity of depressive symptoms, but the reverse was not true (Labouliere et al., 2017). Pre-treatment severity of depressive symptoms led to stronger therapeutic alliances as rated by independent observers, but the adolescents having worse depressive symptoms than anxiety symptoms were perceived as having weaker early alliances by the therapists (Levin et al., 2012).

#### Trauma and Post Traumatic Stress Disorder

Girls built early alliances more easily than boys, and it was the strength of the alliance that led to greater improvement in internalising symptoms than externalising symptoms although both improved and the symptom change was not mediated by the change in alliance (Zorzella et al., 2015).

There was a significant positive progression in both the therapist and child ratings throughout therapy with the ratings of the child changing significantly from session 3 and session 8 to the final session (Zorzella et al., 2017).

Treatment expectancy and collaboration did not influence treatment outcome although it influenced the working alliance parent-carer working alliance affected treatment outcome (Kirsch et al., 2018).

The carers alliance rating was the highest in comparison to the child and therapist rating. Although concordance was low between the therapist and carer with respect to ratings, the alliance contributed to the reduction in symptom severity (Loos et al., 2020).

Valadez-Sanchez (2017) concluded from her study that cultural modifications of TF-CBT exerted a positive influence on the therapeutic alliance ratings of patients and carers.

#### Attention Deficit Hyperactivity Disorder

The results showed that although alliance was significantly higher in those engaged in CBT-PML (Structured CBT), it did not translate into better treatment outcomes and did not play a role in symptom improvement (Boyer et al., 2018).

#### Eating Disorder

The study showed that the internal consistency of the ACF (psychometric scale being evaluated) was high and therapeutic adherence and alliance were excellent with alliance clients having a positive impact on loss of control (bingeing) of patients but not on other variables like other BED disorder symptoms, mood symptoms, and treatment expectation (Puls et al., 2019).

#### The Autism

Child-therapist alliance was a significant predictor of post-treatment anxiety scores, and the parent-therapist alliance was only a marginally significant predictor (Klebanoff et al., 2019). In autism spectrum disorder (ASD), the intelligence quotient of the child positively affected task collaboration late in therapy and was directly related to emotional regulation, child emotional lability, externalising symptoms, and therapist rated the severity of Autism, which predicted emotion regulation (Albaum et al., 2020).

#### Carers

It was observed that the stronger the parent–therapist alliance, the better was the treatment outcome in OCD (Keeley et al., 2011).

In older youths, there was a significant and positive relationship between parent alliance and change in symptom severity (Anderson et al., 2012).

#### Internet-Based Cognitive Behaviour Therapy

The compliance with agreed treatment goals was not found to be adversely affected by using online intervention and in fact was quite the contrary, in that more than 80% completion rate was achieved (Carpenter et al., 2018).

In the study that compared face-to-face and online intervention of the same treatment modality, it was found that there was no difference between the two in terms of alliance ratings. Despite this, the ratings of parents attending the clinic were significantly higher than the parents of children attending online CBT, where there was no face-to-face contact and minimal therapist contact. Furthermore, the study found that the age of the child (older the child) was a factor in the outcome being predicted by a stronger alliance (Anderson et al., 2012).

Higher levels of computer comfortability were associated with increased treatment response along with self and clinician-rated severity of depressive symptoms (Stjerneklar et al., 2019).

#### Demographics

Increase in alliance influenced involvement in therapy and was significantly directly proportional to age, i.e., the older the child, the stronger was the involvement (McLeod et al., 2014).

In older youths, there was a significant and positive relationship between parent alliance and treatment outcome in that stronger parent alliance led to better treatment outcomes from pre-treatment to 6 months post-treatment but this was not significant for younger youths (Anderson et al., 2012).

Girls were found to establish stronger alliances early on than boys in trauma therapy (Zorzella et al., 2015). However, another study found that neither age nor gender had a significant effect on alliance in anxiety disorder (Chu et al., 2014). The female gender predicted a better treatment response to online intervention in anxiety disorders (Stjerneklar et al., 2019).

#### Interpersonal Relationships

The stronger the interpersonal relationships and support they received from these relationships, the stronger was the alliance in children with anxiety and depressive disorder (Levin et al., 2012).

#### Therapeutic Alliance Measures

The TASC, TPOCS, and VTAS used in children to measure therapeutic alliance had high internal consistency and inter-rater reliability (McLeod et al., 2017).

### D-Discussion

#### Professional Orientation and Implications

A lack of diversity was observed in the orientation of the professional leading the studies. All the authors were Psychologists in orientation. Child Mental Health operates through a multidisciplinary team, which means that various professionals are involved in the care of the child and family and is aimed at improving performance, adding to the pool of expertise, and enhancing the effectiveness of the interventions offered to patients (Roberge and van Dick, 2010). “A Critical Moment in Psychiatry: The Need for Meaningful Psychotherapy Training in Psychiatry” (Dr. Renato Alarcón, February 2020, The Psychiatric Times) emphasises the need for training in psychological therapies for Psychiatrists and this lack was evident in the studies.

#### Geographic Distribution of Studies

More than half the studies were conducted in the United States of America alone with North America conducting more than half of the studies. The United States of America has undertaken vigorous research in psychotherapy following World War II (Lloyd, 2015), and this literature review publication may be a reflection of that aspect, in which, although beneficial to research, results have to be cautiously applied universally due to differences in demographics limiting the generalisability to other regions. Thus, this may cause unintended harm if not critically reviewed for suitability and applicability to other regions due to philosophical or cultural differences (Christopher et al., 2014; Sidhu, 2017).

#### Language

English was the dominant language of publication which could have possibly led to the absence of studies in other languages (Neimann Rasmussen and Montgomery, 2018). Therefore, both the EBSCOHost and Google database search for this review was set to include abstracts in languages other than English. These were then translated using Google translate, thus minimising language bias.

#### Publication

Although the consideration of the impact factor in literature review is controversial, the objective analysis of publication bias is essential in a literature review to consider the results obtained (Ayorinde et al., 2020). The wide range of the impact factor of publications included in this literature review would appear to mitigate the publication bias and the inclusion of one publication that did not have an impact factor (Valadez-Sanchez, 2017). Only published studies were eligible to be included in this review but the origins of the studies stemmed from research trials to research undertaken as part of dissertation thesis (Keeley et al., 2011; Levin et al., 2012; Valadez-Sanchez, 2017) and this helped reduce bias (Ahmed et al., 2012).

#### Bias

There was, approximately, an equal number of cohort and randomised control studies. However, there was an absence of blinding during randomisation in one study (Klebanoff et al., 2019). Some studies included missing data from study drop-outs by using software analysis (Marker et al., 2013) to increase the power. This was considered significant bias in a study that evaluated alliance with symptomatology as it has been shown that the quality of therapeutic alliance is predicted to increase engagement in services (Allen et al., 2017). Although this study was evaluating alliance, resultant drop-outs factored in using software analysis could have skewed results and are discussed in limitations. In their study, the plotting of the slope of the changes in the alliance was interpreted in study analyses despite the tail end of the alliance ratings being missing due to dropouts from the study. Although study drop-outs mimic real-life situations, study results should be interpreted with caution, more so in the context of small study samples to begin with, particularly in RCTs (Wood et al., 2004). In other studies, findings were presented only from one arm of the trial, intervention arm, or the treatment completers (Zorzella et al., 2017; Kirsch et al., 2018). Although bias interpretation is dependent on the study outcome results, it is important to address this within the study so that readers are aware of this (Bell et al., 2013). In one study, trying to understand the time effects on ratings, the authors attempted to simplify highly complex variables and compared the agreement of change in alliance to the outcome a year later (Fjermestad et al., 2016), the outcome of results could have been affected by recall bias due to the passage of time and a shorter recall period than one of 12 months would have been preferable (Althubaiti, 2016).

#### Ethics and Confidentiality

Children and parents were informed that the therapist would not see their results in some studies as mentioned in the study results, and their completed rating scales were dropped into a sealed box, perhaps due to how the confidentiality assurance increased the accuracy of response (Carlisle et al., 2006; Kaiser, 2009). Younger children can be biased to score their therapist or their sessions favourably for fear of upsetting their therapist. Therefore, it was essential to assuage their worries by ensuring the confidentiality of their ratings (Creed and Kendall, 2005).

One study provided families with a $15 moratorium (Klebanoff et al., 2019), although the rationale for doing so was not discussed in the study. Although ethics guidelines do not prohibit payments to study subjects, these financial issues should be considered in the context of “undue influence” and “coercion” (Williams and Walter, 2015).

#### Study Setting and Sample Size

As indicated in the description of the studies table, the studies were set in the context of research settings, which are very different from actual clinical. Although difficult to replicate in a clinical setting, the interpretation of results should be considered with respect to this observation (Whitehead et al., 2019) as the researchers probably did not face similar workplace-based challenges faced by therapists from a clinical and administrative perspective (Ehrenreich-May et al., 2011). McLeod et al. (2016) found that the highest alliance ratings can be found in research settings when compared to the clinic and usual settings. Caution should be exercised before generalising these results. The validity of the effect sizes is difficult to comment on because the sizes of the sample were small (Slavin and Smith, 2009). Sample sizes in this review are an important consideration in generalising the results to the wider population as the sample size ranged from 4 to 151 subjects. Anxiety-based disorders are more common in children, so are referrals to treatment centres and this study reflects this, in that most of the studies were conducted in the context of anxiety-based disorders. Therefore, study outcomes from this review could be applicable to these disorders and general clinical settings in the context of interventions offered, even when the sample size is not too large (Faber and Fonseca, 2014). In the study by Klebanoff et al. (2019), the typically developing (TD) with anxiety group was selected through requests to schools, children referred through school psychologists who received letters regarding the study, and referrals accepted to the medical centres. On the other hand, ASD, with anxiety group subjects, were taken through referrals to the medical centres in addition to referrals from regional centres, school staff, and parent support groups, which could have led to a referral bias (Al-Hasan et al., 2011).

#### Demographics

Although the collective age ranged from 5 to 18 years, the mean was about 12 years, and application of these results to all age groups should be done with keeping this in mind. Findings suggest that developmental age matters in collaborative tasks set at different times during sessions. This finding, considered by the therapist, will probably lead to improvement in therapeutic outcomes (Zandberg et al., 2015). Differences in rater assessments of alliance whether it is the child, therapist, or parent led to associations of child factors and alliance and were not an indicator of age (McLeod, 2011; McLeod et al., 2017). There was a predominance of the female gender in the anxiety-based disorder studies, which reflects the prevalence of anxiety disorder (McLean et al., 2011). Despite this, the study that set out to determine if gender was a predictor and in which females showed more improvement over time, only had 14 males in comparison to 51 females. This outcome should be treated with caution (Puls et al., 2019). The other study which found gender disparity in a study of TF-CBT found that girls developed a positive alliance early on in therapy in comparison to boys, however, this again should be interpreted in light of the low power and disparity in the number of girls and boys (Zorzella et al., 2015). The study also did not find a difference in gender with respect to internalising or externalising symptoms (Zorzella et al., 2015), in concurrence with another study which demonstrated that emotional and sexual abuse in combination led to higher externalising symptoms in boys (Mills et al., 2013). The generalisability of the studies is restricted by the socio-economic makeup as the collective sample was middle-class or higher socioeconomic class. The overwhelming dominance of Caucasians in the studies does not lend to generalising the study findings to a wider ethnically diverse population.

#### Medication and Comorbidities

Although one study (Chu et al., 2014) had mentioned that SSRI’s medication did not adversely affect the alliance, this finding was important as 42% of the study subjects were on medication although a study in adults found that there is a weak association between medication adherence and therapeutic alliance (Chang et al., 2019). Medication and effects on alliance need to be studied further as most of the studies had included stability of medication dose as a pre-requisite for study inclusion, making no other observations in study results. This is particularly relevant as one meta-analytic study demonstrated that there is mixed evidence for the relationship of medication to CBT (Hofmann et al., 2012). It is evident from the description of participants, that the studies included patients who were on medication (Levin et al., 2012; Chu et al., 2014; Boyer et al., 2018; Klebanoff et al., 2019). However, medication was not studied as a mediator in the therapeutic alliance, although the number of patients on medication was as high as 83% in one study (Boyer et al., 2018). Hence, medication was a recommended variable for future research. It would also benefit to understand how therapeutic alliance could in turn increase patient compliance if medication is indicated (Chang et al., 2019).

#### Study Aims and Methodology

Studies reviewed were methodologically sound for the objectives they set (Higgins and Green, 2011).

#### Innovations in Cognitive Behaviour Therapy

A wide variety of innovative CBT modalities have been used, and it is outside the scope of this review to compare the various interventions in terms of therapeutic alliance outcomes however it is important to note the variety of innovative approaches utilised.

#### Therapeutic Alliance in the Context of Mental Health Disorders

A variety of mental health disorders have been considered in the various studies with Anxiety based disorders dominating. This is reflective of the prevalence of mental health issues in child and mental health where anxiety-based disorders are more prevalent than others worldwide (Merikangas et al., 2010). Although in the United States, it is ADHD that is most commonly diagnosed as per the Centers for Disease Control and Prevention website.

Therapeutic alliance (TA) was a predictor of a subsequent change in OCD symptoms after controlling for a prior change of symptoms (Keeley et al., 2011). The study found that post-treatment improvement in symptomatology was predicted by the therapeutic alliance as in a previous study by Kazdin et al. (2005). Their finding that exposure work did not adversely affect alliance is hoped to allow clinicians to approach exposure work with this assurance rather than with reservations about introducing this vital task in anxiety work (Keeley et al., 2011). A more supportive, empathic, and encouraging approach was proposed to reduce the difficulty in engaging in collaborative tasks with the therapist (Keeley et al., 2011). When the therapeutic alliance was measured at various points during therapy, there was evidence of changes in alliance ratings. This demonstrates that alliance shifts during therapy, and it follows that it is predominantly the role of the therapist to be aware of this especially when there is a disruption in the alliance as the alliance is found to be a significant predictor of the treatment outcome (Ardito and Rabellino, 2011). Higher anxiety levels positively impacted on therapeutic alliance and this observation in this review was contrary to a previous study (Shirk and Karver, 2003). An important recommendation from this study was that therapists should spend more time initially with clients and parents establishing rapport to help promote alliance (Keeley et al., 2011). This was based on their observation that there was a positive shift in alliance in the first five sessions of therapy despite the introduction of exposure tasks also evidenced in another study (Keeley et al., 2011; Chu et al., 2014).

The early therapeutic alliance was found to predict future depressive symptomatology (Marker et al., 2013; Chu et al., 2014; Labouliere et al., 2017) with alliance predicting the disorder symptoms rather than vice-versa, which are helpful to the therapist in incorporating tasks that enhance alliance and in turn help to alleviate the symptoms post-treatment (Freire et al., 2014).

With respect to anxiety disorders, it was found that gender, age, or the use of SSRI did not affect the therapeutic alliance, although the authors attributed this to the therapist characteristics and ability to accommodate a wide range of client characteristics (Chu et al., 2014).

In trauma work, the ability of a young child to verbalise how they feel and the trust they need to build with a therapist is critical to effective therapy (Scheeringa, 2011). Working with traumatised children would require acute awareness on the part of the therapist to be aware of the transference phenomenon and therefore the therapy would benefit from the supervision of the therapist (Ellis et al., 2019). Alliance ratings were found to get progressively more positive especially when trauma work was introduced in the intervention and this is a significant finding especially when trauma could adversely affect the trust of the child in a caregiver or therapist (Zorzella et al., 2017). This was in contrast to other study results that observed that trauma narrative could significantly affect their relationship with the therapist (Lawson, 2009). Importantly it is recommended that therapists monitor countertransference in therapy (El Husseini et al., 2016). It was observed that although the alliance ratings of children and parents were aligned and treatment expectancy significantly increased these ratings, this had no bearing on the improvement of symptomatology (Kirsch et al., 2018). There was further support for exposure work and CBT techniques in terms of not affecting the alliance. In the study exploring the cultural perspective of trauma therapy, the findings supported the view that incorporating cultural themes in the intervention allows for more effective collaboration with the therapist (Valadez-Sanchez, 2017). In one study regarding PTSD and TF-CBT, alliance ratings of therapists were lower compared to caregivers. This was attributed to subjective opinions and to the fact that therapists have a comparatively larger base to evaluate alliances in comparison to caregivers who are only rating a single therapist and have no one else to compare the therapist to Loos et al. (2020). In view of these observations, it was recommended that therapists should pay very close attention to micro-processes during therapy as they found evidence of good to moderate agreement between caregiver and therapist (Loos et al., 2020).

Engagement coping style positively influenced therapeutic alliance but not the initial status of the alliance (Chu et al., 2014) in contrast to the study that engagement coping was related to both early alliance and later alliance (Whitehead et al., 2019). Involvement was significant for older children but not for younger ones, and this, indirectly, could be possibly due to the developmental age of a child and the ability to engage with adults. However, it could also be explained by the dependence of the younger children to be guided by carers and, therefore, establishing a therapeutic alliance with carers is equally important in effective treatment interventions in children (Accurso and Garland, 2015). It was felt that parents who have an opinion that their child is not bonding or feel the therapist not working collaboratively would resist the intervention (Marker et al., 2013). Therefore, initial efforts have to focus on building a collaborative, empathic relationship to increase the chances of intervention success in anxiety disorders in children (Hudson et al., 2014). Although early alliance did not predict the extent of patient involvement in therapy, an improvement in involvement helped build stronger alliance later in therapy. Thus, revealing complexities in the alliance process in children with anxiety (McLeod et al., 2014), and that this could be mediated by the observation that when clients show interest, there is also a reciprocal involvement from the therapist (McLeod et al., 2014; Daniels et al., 2017).

This review lends support to the theory that parent alliance ratings predict positive treatment outcomes. Children do not attend child and adolescent mental health clinics on their own, they are either referred by an adult in their life and generally, it is the parents who have to even encourage the children to attend (Keeley et al., 2011; McLeod, 2011). It is the carers or teachers or a significant relationship in the life of a child that needs to be sought if interventions are to be more effective (Keeley et al., 2011) in the paediatric population with OCD. Parents act as co-therapists during exposure therapy and it is the empathy and understanding from parents or therapists that are hugely contributory to recovery from this debilitating condition for many children (Dowell and Ogles, 2010; Haine-Schlagel and Walsh, 2015). A meta-analysis of youth psychotherapy in general, and not specifically individual CBT, demonstrated that the treatment outcome was associated with the alliance and that the effect size of the alliance ratings of the youth and parents was nearly identical (McLeod, 2011).

Children with autism have social, behavioural, and communication difficulties (Frye, 2018) which can impact alliance formation. It is noted from the study results that early bond and task collaboration had no effect on alliance but did later in the therapy (Albaum et al., 2020). Autism renders these children extremely sensitive to their often rigid views and perception (Mazefsky and White, 2014), and the study findings indicate that emotion regulation is influenced by therapeutic alliance and child reported emotion response inhibition which tended to improve the therapeutic bond, thus influencing the therapeutic process (Albaum et al., 2020). Child readiness to participate did not influence therapeutic alliance, which has huge implications for clinical practice as very often, the refusal to engage of a child may be perceived as a predictor of failure of intervention planned in ASD and can lead to distress for all concerned (O’Nions et al., 2018). Although the study participants were mainly male, this was representative of the general prevalence of Autism which is predominantly in males. Boys are four times more likely than girls to be diagnosed with Autism^1^. The other study by Klebanoff et al. (2019) observed that children with ASD may not accurately self-report due to a lack of insight and ability to communicate thoughts and feelings accurately. Therefore, they would be less reliable reporters of measures of the alliance, and although it was observed that children with anxiety and ASD established poorer alliances than those without ASD. This would be indicative of the poorer social skills of children with ASD rather than directly related to the therapeutic alliance (Frye, 2018). However, in this review, the hypothesis that parent alliance was more reliant than child alliance in predicting outcome was not confirmed in youth with ASD (Klebanoff et al., 2019), which was in contrast to previous studies (McLeod, 2011) where both child and parent ratings were similar in predicting outcome. The child-therapist alliance was more strongly predictive of treatment outcome even as there is perceived difficulty in gaining initial trust in children with ASD, these children need time to develop a rapport with their therapist before engaging in the intervention and can eventually establish alliance (Klebanoff et al., 2019). Positive late alliance ratings need to be viewed with caution as improvement in symptomatology may be influencing alliance rather than vice versa (Shirk and Karver, 2003). Independent observer rating was recommended to reduce this bias (Albaum et al., 2020).

Although the alliance was higher in those patients with ADHD who received CBT focussed on planning skills, this did not translate into improved symptomatology post-treatment and instead it was the degree to which collaborative tasks were carried out that predicted treatment outcome (Langberg et al., 2011, 2016; Boyer et al., 2018). The observation that the therapist needs to step up with innovation, empathy, and consideration when therapy is less structured was in agreement with previous study findings which observed the active involvement of a therapist influenced the treatment concordance in clients (Naar and Safren, 2017; Boyer et al., 2018). The less structured therapy needed more focus or concentration and may explain the results of this study (Boyer et al., 2018).

Boyer et al. (2018) did not set out to link alliance and treatment outcome as in previous studies (Horvath et al., 2011) but they examined client characteristics like existing positive relationships and observed that youth with an experience of positive relationships engaged more strongly with their therapists. It may be that having a template for a trusting relationship assists in collaborating with the therapist (Crits-Christoph et al., 2019).

In eating disorders, higher levels of the treatment expectation of the adolescent were significantly related to lower adherence. This occurrence is attributed to the possibility that the perceived lower input from the therapist due to their own higher expectation of treatment might have led to decreased engagement in the intervention in part due to their demand for more individualised treatment plan from the therapist leading to lowered adherence to the intervention protocol (Puls et al., 2019).

#### Psychometric Measures

Therapeutic alliance was measured using the Psychometric scales. Originally implemented for measurement of a single perspective, they have been modified over time or extended to include measures that had not been previously included. While some incorporate specific constructs of an alliance like the Working Alliance Inventory (WAI) and California Psychotherapy Alliance Scale (CALPAS), others like VTAS have a broader base of sources of the alliance perspectives (Ardito and Rabellino, 2011). Objective raters were used in the studies by Labouliere et al. (2017), Kirsch et al. (2018), and Albaum et al. (2020). Objective raters were not used in the studies by Marker et al. (2013) and Fjermestad et al. (2016), and their findings contrast with the studies using objective raters. Observer-rated alliance measures decreased over time (Whitehead et al., 2019). It also made comparisons between studies more difficult even when rating the same outcome. A therapist answering questions like “The child likes spending time with you, the therapist” was simplistic and highly subjective even as they interpreted essential subjective opinion of both child and therapist (Shirk and Karver, 2003). The dual slope method (Chu et al., 2014) was a good visual depiction of the changes in alliance and helped depict alliance as a continuum, and a “shifting” process (Safran and Muran, 2001).

#### Working Alliance Inventory

The WAI is a 36-item measure of the quality of the TA in adult psychological treatment (Keeley et al., 2011) and was adapted as a suitable assessment of the parent–therapist alliance in child psychological treatment (Kazdin et al., 2005). Items are anchored on a 1–7 scale and the WAI is a widely used measure, with proven reliability and validity (Horvath and Bedi, 2002). The construct validity of the WAI-S was used in the internet-based therapy for children with anxiety disorder and proved to have strong psychometric properties in assisting the measure of alliance and was proposed as a reliable measure in internet-based interventions (Anderson et al., 2012) but questions were raised if their answers were influenced by their carers/parents at home.

The TPOCS-A showed convergent validity with independent self and observer-report alliance instruments (Fjermestad et al., 2016; McLeod et al., 2017). Although the scales were validated in one of the studies, McLeod et al. (2017), these measures fail to adequately capture the emotional element when rating, especially in children.

There were observed differences between therapist and observer ratings, and this was attributed to variability in considering factors differently when alliance ratings were completed (McLeod et al., 2014). As observed in their study, many items in the TPOCS-A, had to be rated and this perhaps led to these items being interpreted differently (Chu et al., 2014; McLeod et al., 2014).

In evaluating rating scales in children with anxiety disorders, there appeared to be issued with convergence regarding observer-therapist ratings and younger children vs. adolescents, demonstrating low convergence with children but strong with adolescents with respect to TASC-C (McLeod et al., 2017). The affective and cognitive development of younger children could impact their understanding of the meaning of “alliance” was offered as a possible explanation for this finding (McLeod et al., 2017).

#### Therapist Context

It appears that rather than patients, clinicians themselves worry about therapeutic alliance (Lopez et al., 2019). Client perception or interest in alliance tended to be underestimated by therapists which could be because of the lack of awareness or misinterpretation of client symptoms (Zandberg et al., 2015). The study conditions were unique in that all the therapists had supervision although these research conditions do not translate into real-life situations and whether supervision allocated to trainee therapists was a factor in the study conclusion and needs to be explored further (Levin et al., 2012; Asarnow and Miranda, 2014). Many of the therapists were trainees and in the early stages of training which could have influenced their perception of alliance (Levin et al., 2012). It is, however, unclear from this literature review if that is a confounding factor in many of the study outcomes and could be considered for future research (VanderWal, 2015). On the other hand, in the absence of supervision or reflective practice, it is quite difficult to be aware of treatment processes occurring within the session for those in training in CBT (Bennett-Levy et al., 2009). Therapists working with children need to be aware of attachment styles of children or interpretation of observed attachment in therapy as the absence of this awareness might affect the evaluation of therapy and its outcomes and needs to be considered particularly in younger children (Cassidy et al., 2013). An awareness of psychopathology when working with children with mental health issues is essential for the therapist, it leads to a better understanding of presentation in therapy, for example, a depressed child may exhibit either irritability or negative affect, and awareness of this would assist the therapeutic process by bringing about effective collaboration in tasks to target the symptoms (Halder and Mahato, 2019).

#### Social Constructs and Relationships

Positive experience of trusting relationships outside of therapy translated into stronger alliances early on in therapy (Levin et al., 2012). Particularly, TF-CBT work in children who had suffered trauma and a break in trust. The finding was consistent with the attachment theory that “children use their relationships with caregivers to create internal working models, which carry over to other relationships, including the therapeutic relationship,” (Bowlby, 1973). The social relationship aspect in depression is important as one of the first signs to be noted is a child withdrawing socially from existing relationships (Loades et al., 2020). Studies within this literature review have alluded to templates for social relationships. Relationships to authority figures in the context of establishing an alliance with the therapist (Levin et al., 2012; Whitehead et al., 2019), and to observe that knowledge of the relationship variables further facilitated compliance and collaboration with the therapist (Whitehead et al., 2019).

#### Internet-Based Cognitive Behaviour Therapy

Although one study demonstrated that therapeutic alliance can be established effectively through online therapy in adults (Cook and Doyle, 2002), others have contended this claim (Rochlen et al., 2004). The study by Anderson et al. (2012) was robustly conducted even though it lacked socioeconomic diversity, they observed that therapeutic alliance can be established through online therapy even as parents attending face-to-face therapy rated alliance higher than parents of children who attended online therapy. This is significant in the current times of telemedicine due to the COVID-19 crisis. With the younger generation being technology savvy, their adherence to treatment could also increase and with the constraints of time, online therapy would be great for parents and carers as evidenced in the studies (Carpenter et al., 2018; Klebanoff et al., 2019; Loos et al., 2020). Although the sample size was small, these studies are contrary to the general acceptance that “the therapeutic alliance can only be established face-to-face” and is an exciting finding of this literature review which is in concordance with other opinions which state that alliance can be established without face-to-face contact (Berger, 2017; Wind et al., 2020). There was observed discrepancy in alliance only at follow-up and only at a year of follow-up, which was considered not as important as the direction of change of alliance perspective between therapist and client in an internet-based CBT study (Stjerneklar et al., 2019). The same study (Stjerneklar et al., 2019) observed no relationship between alliance and outcome but called for alliance measurements to be undertaken at multiple points rather than one point as had been done in their study (Stjerneklar et al., 2019). Children with more severe baseline anxiety symptoms benefitted as much if not more from the internet-based CBT by demonstrating greater improvements in symptoms and this has significant implications for clinicians when considering internet-based treatment exclusion for those with more severe anxiety symptoms (Stjerneklar et al., 2019). The pilot study of TMH-FCBT in anxiety disorder produced favourable results with a low dropout rate and high treatment satisfaction (Carpenter et al., 2018).

### E-Limitations

#### Methodology

RCT compared children with anxiety with ASD and those without ASD. It is known that these two cohorts share phenotypes, similar developmental, behavioural, and cognitive profiles (Wiggins et al., 2019) which overlap and could have confounded results in the study, this observation recommended further studies to compare alliance in ASD with other disorders (Klebanoff et al., 2019). Statistical significance was possibly compromised by small sample size and it was an issue in the trials by way of introducing sampling bias leading to the reduced power of studies (Anderson et al., 2012; Levin et al., 2012; Chu et al., 2014; McLeod et al., 2014; Labouliere et al., 2017; Zorzella et al., 2017; Carpenter et al., 2018; Kirsch et al., 2018; Albaum et al., 2020; Loos et al., 2020). The cohort studied was recruited from a larger study evaluating therapist adherence and these findings need to be applied with caution to recommendations to clinic situations (Kukull and Ganguli, 2012; Albaum et al., 2020). Although their sample was taken from a larger RCT, that sample was demographically representative of the local population (Boyer et al., 2018). The two forms of CBT, SFT, and PML used to compare in the groups were similar in many aspects and it would be useful to introduce another comparison intervention to study alliance and treatment outcomes in ADHD especially as one study has shown similar outcome whether CBT was used or not, albeit in adults (Boyer et al., 2018; Corbisiero et al., 2018). Although their study demonstrated that exposure work did not affect therapeutic alliance in OCD, it was noted that causal association could not be derived from the study as the study did not change any of the variables of therapeutic alliance and potential moderators of the alliance could not be measured due to the small size of the study sample (25 participants) (Keeley et al., 2011), and caution needs to be exercised in the application of the study results from a small sample (Leppink et al., 2016). Exclusion of drop outs from the study could have been due to interpersonal factors and omitting them from the study analyses could potentially have skewed the results (Levin et al., 2012). To truly understand the moderating variables of the alliance, in OCD, it was recommended that child-rated measures were collected (Zorzella et al., 2015), due to this being a limiting factor of their study. Using multilevel models limited the statistical power in the study (Whitehead et al., 2019). Recruiting samples from the urban areas in a study setting with respect to Telemental health intervention requires cautious interpretation of the finding that it is possible to establish a therapeutic alliance when most rural areas are still lagging in terms of good internet connectivity which is essential to smooth treatment intervention, so sampling bias has to be considered in this study as all the participants were from the urban area with good connectivity (Chou et al., 2016; Carpenter et al., 2018).

#### Demographics

The gender effects cannot be generalised from the study with ASD in general as there were more males (91.7%) than females in the study. Although previous studies in ASD did not demonstrate gender differences (Albaum et al., 2020), one study compared CBT with treatment as usual (Kerns et al., 2018). Gender was also noted as a bias in the study by Loos et al. (2020) with 53 females and 23 males. The study that included seven 20-year-olds due to delay in the start of treatment controlled for age in the sub-analyses and found no effects due to living conditions on alliance ratings (Puls et al., 2019). The motivation to change in children and its impact on the therapeutic alliance was not considered in their study and hence was noted as a limitation in the study of the therapeutic alliance (Marker et al., 2013). Sampling bias with respect to ethnicities and socioeconomic status was noted in studies due to lack of diversity in ethnicity and socioeconomic status (Carpenter et al., 2018; Klebanoff et al., 2019; Whitehead et al., 2019).

#### Therapy Factors/Psychometric Rating

Only observer ratings were used (Albaum et al., 2020) and it was recommended that using multiple raters would limit this bias as alliance ratings can be influenced by the source of ratings (Crits-Christoph et al., 2011). This was also a limitation in another study in which the authors postulated that multiple informant perspectives would help in more clear perspective of the alliance by eliminating the lack of awareness of implicit factors like interpersonal functioning and attachment in therapy (Levin et al., 2012). Although questions were raised about the use of TPOCS-A in their study due to discriminant validity between involvement and alliance, other studies have shown convergent validity during its’ use to rate both observer and self-ratings of alliance (Fjermestad et al., 2016; McLeod et al., 2017; Loos et al., 2020). Although concerned that observations of parents ratings were all “good” or “excellent,” in their study, the authors felt that the parents were eager and hoping for the recovery of their children from debilitating symptoms of PTSD, would have voiced any degree of concern they had and therefore the ratings were acceptable for study analysis due to the consistent ratings across the cohort, although it was felt that introducing independent raters would give more definitive answers to this observation (Zorzella et al., 2015, 2017). It was observed that repeated measures could have affected the outcome of analysis as their study showed no effect of TE on treatment outcomes (Kirsch et al., 2018), in contrast to another study that did, but that study was in OCD (Lewin et al., 2011). Pre-treatment to treatment changes of depressive symptoms (Gamble et al., 2010) were not assessed in the alliance study in depressed children. Waitlist times were different for different study subjects, which could have also led to variability in the pre-treatment gains and given that the changes in therapeutic alliance magnitude were small (Labouliere et al., 2017). Therapists were aware of the sessions being coded to measure alliance and this could have introduced bias within-session in how they were rated (Levin et al., 2012). The absence of setting fixed sessions led to therapists choosing anywhere between 16 to 20 sessions indirectly, with only 26% reaching session 18, making the trajectory model less reliable (Chu et al., 2014). The audio and video recordings of the sessions were rated variably by independent raters which affected the validity of the results by less accurate assessment of the therapy process in sessions (Hudson et al., 2014). The accreditation and training levels of therapists have to be considered before extrapolating results to clinical settings as extensive training and supervision was given to therapists even though studies (McLeod, 2011; Goldsmith et al., 2015) did not find that this affected therapeutic alliance. It is useful to reflect on and consider the therapist’s age and stage of training with respect to reporting on alliance measures (Zandberg et al., 2015). Including qualitative and quantitative measures were considered as more accurate assessments of alliance than quantitative measures alone (Anderson et al., 2012). Measurements over different points in time were indicated for future studies (Anderson et al., 2012; McLeod et al., 2014; Fjermestad et al., 2016). Fjermestad et al. (2016), also noted that the alliance ratings were through using psychometric measures used in the United States and not in Norway and noted that therapist competence could affect ratings. Therapists acting as raters was noted as a limitation although attempts were made to reduce this by blinding raters to patient details and through objective rating and supervision (Puls et al., 2019).

#### Mental Health Disorders

The study was conducted in high functioning children with ASD and does not intend to generalise the study findings to those with lower intellectual capabilities and ASD as children with lower capabilities differ in many respects (Srivastava and Schwartz, 2014; Klebanoff et al., 2019). It is also known that ASD impacts social functioning which could have influenced the alliance outcome and, therefore, further studies on larger cohorts are recommended (Bishop-Fitzpatrick et al., 2017; Klebanoff et al., 2019). Study outcomes conducted in one mental health disorder were not considered as generalisable to other mental health issues in terms of applicability of therapeutic alliance in CBT (McLeod et al., 2014, 2017; Carpenter et al., 2018).

## Conclusion and Recommendations

This review supports the view that individualised treatment plans are essential for all patients. The theories of attachment of Bowlby (1988) appear to be as relevant in the current understanding of alliance as they were more than half a century ago and need to be researched further in children to gain further insight in understanding alliance concepts. The concept of the parent-carer alliance with the child during CBT in terms of the factors influencing parent-carer alliance with the therapist is one that can be explored in future research studies. This alliance with their children in many interventions, particularly in those of younger age, could effect a change in the treatment outcome of many mental health disorders. However, there was one aspect of child mental health that was excluded as a variable in all the studies and that is “suicidal ideation” or “suicidality” in children with mental health issues, which perhaps need to be included in future studies in enhancing better understanding of this concept in children with suicidal ideation. Another variable that could predict therapeutic alliance is the use of medication which needs further research in understanding its influence on therapeutic alliance. Including these two variables in future research studies would help clinicians working with children in preventing fatal outcomes and inform prescribing of medication in children.

Therapeutic alliance scales, which were initially devised to evaluate therapeutic alliance in adults, were used in the studies and one of the aims of this review was to determine if these were adequate to capture the three pillars of the therapeutic alliance, namely, “congruence, empathy, and unconditional positive regard,” as posited by Rogers (1951) in children from a developmental aspect. However, it was unclear from this review, if the rating scales adequately capture developmental influence in the evaluation of therapeutic alliance, and further research in this area is indicated. Although the majority of the studies were undertaken in ideal research settings, extrapolating them to real-life situations and conducting research in these settings will help to either consolidate or bring about new inferences. The multi-rater assessments, multi-point in time assessments, parent-child therapist assessments demonstrate the complexity of understanding therapeutic alliance in child and adolescent mental health service. When teacher ratings are thrown in the mix, the potential to harness all the assistance in helping the child surmount mental health challenges is breath-taking but equally from a research perspective very challenging which may in part explain the contradictory findings in the literature review.

The literature review reveals that although gaps exist in current research, it is essential to acknowledge the progress being made to address these gaps. Even though many of the studies were underpowered, the results can still fuel further Research by providing insight through the outcome and limitations described in the studies. Working with parents to collaborate and engage them when treating the more severely affected children, the outcomes of studies demonstrate that Internet-based therapy should not only be reserved for mild severity of illness but can also work in severe mental illness especially in debilitating social anxiety, albeit to give a chance to the patient to start making incremental gains by establishing communication with the therapist is an interesting finding from this review. This comes with the caution that when choosing patients for internet-based intervention, one needs to ensure good internet connectivity as it would be self-defeating to allocate internet-based CBT to a patient with no or poor connectivity.

Although all the questions could not be answered, future research could aim to address previous theoretical frameworks of understanding the concept of therapeutic alliance and its’ clinical application to Child and Adolescent Psychiatry and it is undeniable that the studies discussed in this review will transform and enhance our understanding of our ability to transform child mental health outcomes more positively through therapeutic alliance-building measures.

## Data Availability Statement

The original contributions presented in the study are included in the article/Supplementary Material, further inquiries can be directed to the corresponding author/s.

## Author Contributions

The author confirms being the sole contributor of this work and has approved it for publication. This project has not been submitted either in part or whole by any other person other than me to any other publication. This project was submitted by me for the purpose of meeting the Dissertation course requirements for the Masters in Cognitive Behavioural Therapy of the Irish College of Humanities and Applied Sciences, Limerick, Ireland (Fernandes, 2020).

## Conflict of Interest

The author declares that the research was conducted in the absence of any commercial or financial relationships that could be construed as a potential conflict of interest.

## Publisher’s Note

All claims expressed in this article are solely those of the authors and do not necessarily represent those of their affiliated organizations, or those of the publisher, the editors and the reviewers. Any product that may be evaluated in this article, or claim that may be made by its manufacturer, is not guaranteed or endorsed by the publisher.

## Footnotes

1
https://www.autismspeaks.org/autism-statistics

## Abbreviations

AAQ: Adolescent Attachment Questionnaire
ADHD: Attention Deficit Hyperactivity Disorder
ADIS-IV-C/P: Anxiety disorders interview schedule, child, and parent version
ADOS-2: Autism Diagnostic Observation Schedule- Second edition
AOCS: Alliance Observation Coding System
ASD: Autistic Spectrum Disorder
BASC-2 PRS: behavioural Assessment System for Children-Second edition
BDI: Beck Depressive Inventory
BED: Binge eating Disorder
BEDA: Binge eating disorder in adolescents
BTPS: Barriers to Treatment Participation Scale
CAMHS: Child and Adolescent Mental Health Service
CAPS-CA: Clinician-Administered PTSD Scale for Children and Adolescents
CASAFS: Children and Adolescent Social and Adaptive Functioning Scale
CBT: Cognitive Behavioural Therapy
CBCL: Child Behaviour Checklist
CCBT: Child focussed CBT
CCPAC-M: Coping Cat Protocol Adherence Checklist-Modified
CDI: Child Depression Inventory
C-DISC: Computerized Diagnostic Interview Scale for Children
CEMS: Children’s Emotion Management Scales
CGAS: Children’s Global Assessment Scale
CGI-I: Clinical Global Impressions-Improvement Scale
CGI-S: Clinical Global Impressions-Severity Scale
CIRS: Child Involvement Rating scale
CIRS-R: Child Involvement Rating Scale-Revised
CPPS: Child Psychotherapy Process Scales
CSQ: Client Satisfaction Questionnaire
CSR: Clinical Severity Rating
CQ-C: Child questionnaire-Child
CY-BOCS: Children’s Yale-Brown Obsessive-Compulsive Scale
DEBQ: Dutch Eating Behaviour Questionnaire
DISC-IV: Diagnostic Interview Schedule for Children for DSM-IV parent version
ERC: Emotion regulation checklist
FACLIS: Family Accommodation Checklist and Interference Scale
FCBT: Family focused CBT
ICBT: Internet Based Cognitive Behaviour Therapy
MASC-P: Multidimensional Anxiety Scale for Children- Child and Parent
MCBT: Multimodal Cognitive Behavioural Therapy
MEIM-S: Multigroup Ethnic Identity Measure-Short
MITI: Motivational Interviewing Treatment Integrity scale
OCD: Obsessive Compulsive Disorder
MSPSS: Multidimensional Scale of Perceived Social Support—Military Peers Items Addition
PARS: Paediatric Anxiety Rating Scale
PTSD: Post traumatic stress disorder
PTSD-RI: Posttraumatic Stress Disorder Reaction Index
PTSS: Post traumatic stress symptoms
RCADS-P: Revised child anxiety and depression scales-parent version
RCMAS: Revised Children’s Manifest Anxiety Scale
RSQ-P: Response to stress questionnaire-parent version
SCAS-C: Spence Children’s Anxiety Scale-Child Version
SCAS-P: Spence Children’s Anxiety Scale-Parent Version
SEQ-SR: Social Experience Questionnaire
SRS: Social Responsiveness Scale
SSRI: Selective Serotonin reuptake inhibitor
STAIC: State Trait Anxiety Inventory for Children
STAIC-P: State Trait Anxiety Inventory for Children- Parent version
TABBS: Therapist Alliance-Building Behaviour Scale
TABBS-A: Therapist Alliance-Building Behaviour Scale-A
TASC: Therapeutic Alliance scale for Children
TASCP: Therapeutic alliance scale for caregivers and parents
TASC-r: Therapeutic Alliance scale for caregivers and parents-revised
TD: Typically developing
TMH FCBT: Telemental Health Family Cognitive Behavioural therapy
TPOCS-A: Therapy Process Observational Coding System-Alliance Scale
TRF: Teacher Report Form
UP-Y: Unified Protocol for treatment of Emotional disorders in Youth.
VTAS: Vanderbilt Therapeutic Alliance Scale
WAI-S-O-R: Working Alliance Inventory shortened observer-rated version
WAI-S: Working Alliance Inventory- Short form.
