# Supplementary material for: Therapeutic Alliance in Cognitive Behavioural Therapy in Child and Adolescent Mental Health-Current Trends and Future Challenges
Source: Front Psychol. 2022 Jan 3;12:610874. doi: 10.3389/fpsyg.2021.610874 (PMC8763013; doi:10.3389/fpsyg.2021.610874)
Supplement: Supplementary file 1 [file Table_1.pdf]

Table 1: Literature review studies

Studies are listed in chronological order of Publication from the publication source from where the article was accessed

Study Title	Author/Authors	Study design	Database	Publication	Peer reviewed Impact factor	Publication date	Service Context	Age range(years)/ Mean/ Standard deviation	Sample size	Clinical condition	Accredited Therapist	Trainee therapist	Supervised Therapist	Intervention	Number of sessions
The therapeutic alliance in the cognitive behavioral treatment of pediatric obsessive–compulsive disorder.	Keeley, M. L., Geffken, G. R., Ricketts, E., McNamara, J. P. H., & Storch, E. A.	Cohort Study	APA PsycInfo	Journal of Anxiety Disorders	Yes 3.07	October 2011	Specialty OCD Clinics	7-17/13.16/2.69	25	Obsessive compulsive disorder	Yes	Yes	Yes	FCBT	14 (60 mins)
Working alliance in online cognitive behavior therapy for anxiety disorders in youth: Comparison with clinic delivery and its role in predicting outcome	Anderson, R. E. E., Spence, S. H., Donovan, C. L., March, S., Prosser, S., & Kenardy, J.	Study 1-RCT Study 2-Cohort study	APA PsycInfo	Journal of Medical Internet Research	Yes 5.05	June 2012	Research Study participants through invitations to health professionals and media publicity	Study 1-12-18/13.69/1.56 Study2-7-18/12.12/2.5	Study 1-73 Study 2-132	Anxiety disorder	No	Registered Psychologist (2 session training in BRAVE)	Yes	Study 1-Face to face BRAVE or BRAVE for teenagers-online<br><br>Study 2-Minimally assisted online CBT-BRAVE for teenagers-online	Study 1- 10 youth,5 parents, 2 boosters at 1- and 3-months post treatment<br><br>Study 2-12 online sessions, no face to face contact online, short midsession telephone contact to assist exposure hierarchy
Interpersonal predictors of early therapeutic alliance in a transdiagnostic cognitive-behavioral treatment for	Levin, L., Henderson, H. A., & Ehrenreich-May, J.	RCT	APA PsycInfo	Psychotherapy	Yes 1.622	June 2012	Child & Adolescent University Mental Health	12-17/15.9/No SD	31	Anxiety disorder + co morbid mental	No	Yes	Yes	Transdiagnostic CBT(UP-Y)	8-21 (50 mins)

adolescents with anxiety and depression.							Programme Clinic			health diagnosis					
The reciprocal relationship between alliance and symptom improvement across the treatment of childhood anxiety.	Marker, C. D., Comer, J. S., Abramova, V., & Kendall, P. C	Cohort Study	MEDLINE	Journal of Clinical Child and Adolescent Psychology	Yes 5.014	January 2013	Child & Adolescent Anxiety disorders Clinic	7.15-14.44/10.19/1.7	86	Anxiety disorder +Comorbid mental health diagnosis (60%)	Yes	No	Yes (individual by accredited Supervisors)	FCBT	16 (60 mins)
Child involvement, alliance, and therapist flexibility: Process variables in cognitive-behavioural therapy for anxiety disorders in childhood.	Hudson, J. L., Kendall, P. C., Chu, B. C., Gosch, E., Martin, E., Taylor, A., & Knight, A.	Cohort Study	APA PsycInfo	Behaviour Research and Therapy	Yes 4.680	January 2014	Child & Adolescent Anxiety Disorders Clinic	6-14/10.71/No SD	151	Anxiety disorder + co morbid mental health diagnosis (88%)	Yes	No	Not indicated	CBT (Coping Cat)	16 (60 mins)
Trajectory and predictors of alliance in cognitive behavioral therapy for youth anxiety	Chu, B. C., Skriner, L. C., & Zandberg, L. J.	Cohort study	APA PsycInfo	Journal of Clinical Child and Adolescent Psychology	Yes 5.014	September 2014	University Based Outpatient Mental Health Clinic	7-17/12.46/2.64	69	Anxiety disorder +Comorbid mental health diagnosis (89.9%)	No	Yes	Not indicated	CBT	16 (60mins)
Client-therapist alliance discrepancies and outcome in cognitive-behavioral therapy for youth anxiety.	Zandberg, L. J., Skriner, L. C., & Chu, B. C.	Cohort study	APA PsychInfo	Journal of Clinical Psychology	Yes 2.16	April 2015	University Outpatient Clinic	7-17/12.43/0.76	62	Anxiety disorder	No	Yes	Yes	CBT	20(60 mins)
Therapist-youth agreement on alliance change predicts long-term outcome in CBT for anxiety disorders	Fjermestad, K. W., Lerner, M. D., McLeod, B. D., Wergeland, G. J. H., Heiervang, E. R., Silverman, W. K., Öst, L., De Los Reyes, A., Havik, O. E.,	RCT	APA PsycInfo	Journal of Child Psychology and Psychiatry	Yes 5.014	May 2016	Multisite Community Mental Health Clinics	8-15/11.4/2.1	91	Anxiety disorder+ comorbid anxiety disorder (90%) + other	Yes	Yes	Yes	CBT	10 (60 mins) Booster sessions 1 & 3 months

	& Haugland, B. S. M.														
The relationship between alliance and client involvement in CBT for child anxiety disorders.	McLeod, B. D., Islam, N. Y., Chiu, A. W., Smith, M. M., Chu, B. C., & Wood, J. J.	Cohort study	APA PsycInfo	Journal of Clinical Ch ild a nd Adolesc ent Psy chology	Yes 5.014	September 2014	Child & Adolescent Anxiety Clinic	6-13/9.58/2.17	31(14 CCBT;17 FCBT)	Anxiety Disorder (Comorbi d mental health diagnosis not indicated)	Yes	Yes	Yes	CBT	12-16 (60 mins)
The relationships between therapeutic alliance and internalizing and externalizing symptoms in Trauma-Focused Cognitive Behavioral Therapy	Zorzella, K. P. M., Muller, R. T., & Cribbie, R. A.	RCT	APA PsycInfo	Child Abuse & Neglect	Yes 2.569	December 2015	Multisite Community based mental health clinics	7-12/9.58/1.6	95	Trauma	Yes	Yes	Yes	TF CBT	10-45(45mins) (4-12 months)
Therapeutic alliance with depressed adolescents: Predictor or outcome? Disentangling temporal confounds to understand early improvement.	Labouliere, C. D., Reyes, J. P., Shirk, S., & Karver, M.	Cohort study	APA PsycInfo	Journal of Clinical Ch ild and Adolescent Psychology	Yes 5.014	July 2017	School based mental health clinics	14-18/15.89/1.25	38	Depressi ve disorder	Yes	Yes	Yes	CBT	12(60 mins)
Therapeutic alliance over the course of child trauma therapy from three different perspectives.	Zorzella, K. P. M., Rependa, S. L., & Muller, R. T.	Randomi sed control trial	APA PsycInfo	Child Abus e & Neglect	Yes 3.310	May 2017	Multisite Community based mental health clinics	7-12/10/No SD	65	Trauma (>72%Se xual Abuse;>7 4% multiple traumas)	Yes	Yes	Yes	TF CBT	17-18(90 mins) (4-12 months)
More than whoever made you suffer: A culturally informed Trauma-Focused intervention for Latina adolescents	Valadez-Sanchez, K. H.	Cohort study	APA PsycInfo	Dissertation Abstracts Internationa l: Section B: The Sciences and Engineerin g	No impact factor	September 2017	School based	16-18	4	Trauma ; PTSD	No	Yes	Yes	Culturally informed TF CBT	16-18 (50 mins)
Observer, youth, and therapist perspectives on the alliance in cognitive behavioral	McLeod, B. D., Southam-Gerow, M.	Randomi sed control trial	APA PsycInfo	Psychologi cal Assessment (Journal)	Yes 3.890	December 2017	University Research Centre	7-15/10.28/1.84	50	Anxiety disorder+ comorbid mental	Yes	Yes	Not indicated	CBT	12(60 mins)

treatment for youth anxiety.	A., & Kendall, P. C.									health diagnosis					
Treatment expectancy, working alliance, and outcome of Trauma-Focused Cognitive Behavioral Therapy with children and adolescents.	Kirsch, V., Keller, F., Tutus, D., & Goldbeck, L.	RCT	APA PsycInfo	Child & Adolescent Psychiatry and Mental Health	Yes 1.642	March 2018	Child & Adolescent Mental Health Clinics	7-17/12.52/2.9	65	PTSD	Yes	No	Yes	TF-CBT	12 (90 mins)
Comparing alliance in two cognitive-behavioural therapies for adolescents with ADHD using a randomized controlled trial.	Boyer, B., MacKay, K. J., McLeod, B. D., & van der Oord, S.	RCT	APA PsychInf o	Behaviour Therapy	Yes 4.5	September 2018	Mental health clinics	12-17/14.54/No SD	69	ADHD (83% on medicatio n)	Yes	Yes	Not indicated	CBT (structured)<br><br>CBT (solution focussed)	10 (45 mins)
Working from Home: An Initial Pilot Examination of Videoconferencing-Based Cognitive Behavioral Therapy for Anxious Youth Delivered to the Home Setting.	Carpenter, A. L., Pincus, D. B., Furr, J. M., & Comer, J. S.	Cohort Study	MEDLIN E	Behavior t herapy	Yes 4.5	November 2018	Multisite	7-14/9.85/1.46	13	Anxiety Disorder	No	Yes	Yes	TMH FCBT	16 (60 mins)
Child social and emotion functioning as predictors of therapeutic alliance in cognitive–behavioral therapy for anxiety.	Whitehead, M., Jones, A., Bilms, J., Lavner, J., & Suveg, C.	RCT	APA PsycInfo	Journal of Clinical Psychology	Yes 2.16	January 2019	University Research Clinic	7-11/8.93/1.64	92	Anxiety disorder + one other anxiety disorder (92%) Anxiety disorder+ ODD/AD HD (36%)	No	Yes	Yes	CBT<br><br>ECBT	10 (50 mins)
Guided internet-based cognitive behavioral therapy for adolescent anxiety: Predictors of treatment response.	Stjerneklar, S., Hougaard, E., & Thastum, M.	RCT	MEDLIN E	Internet Interventio ns	Yes 3.73	January 2019	Centre for Psychologi cal Treatment of Children & Adolescent s	13-17/15.2/1.33	65	Anxiety Disorder +comorbi d mental health diagnosis	No	Yes	Yes	Internet based CBT	14 (60 mins)

Therapist adherence and therapeutic alliance in individual cognitive-behavioural therapy for adolescent binge-eating disorder	Puls, H.-C., Schmidt, R., & Hilbert, A.	RCT	MEDLINE	European Eating Disorders Review: The Journal of the Eating Disorders Association	Yes 3.2	March 2019	University of Leipzig Medical Centre	12-20/14.17/2.77	64	Binge eating disorder	Yes	No	Yes	CBT	20(50 mins)
The therapeutic alliance in cognitive-behavioral therapy for school-aged children with autism and clinical anxiety.	Klebanoff, S. M., Rosenau, K. A., & Wood, J. J.	RCT	MEDLINE	Autism: The International Journal of Research & Practice	Yes 5.879	November 2019	Medical Centre	5-12/10/2 5-12/8/1.6	36 in TD + anxiety 60 in ASD + anxiety	Autistic Spectrum Disorder - Comorbid mental health diagnosis not indicated	No	Yes	Yes (group supervision)	MCBT	12-16 (60-80mins) in TD with anxiety<br><br>16 (90 mins) ASD with anxiety
Predictors and outcomes associated with therapeutic alliance in cognitive behaviour therapy for children with autism	Albaum, C., Tablon, P., Roudbarani, F., & Weiss, J. A.	Cohort study	APA PsycInfo	Autism	Yes 5.879	January 2020	Not indicated	8-12/9.6/1.25	48	Autistic Spectrum disorder	Yes	Yes	Yes	Emotion regulation focused CBT<br><br>(Secret Agent Society: Operation Regulation)	10 (parent and child)
Do caregivers' perspectives matter? Working alliances and treatment outcomes in trauma-focused cognitive behavioural therapy with children and adolescents.	Loos, S., Tutus, D., Kiliana, R., Goldbeck, L.	Randomised control trial	Open Access	European Journal of Psychotraumatology	Yes 3.02	May 2020	Child & Adolescent Mental Health Clinics	7-17/12.6/2.92	76	PTSD	Yes	Yes	Not indicated	TF-CBT	12(90 mins)
